# Relationship between the Ki67 index and its area based approximation in breast cancer

**DOI:** 10.1186/s12885-018-4735-5

**Published:** 2018-09-03

**Authors:** Muhammad Khalid Khan Niazi, Caglar Senaras, Michael Pennell, Vidya Arole, Gary Tozbikian, Metin N. Gurcan

**Affiliations:** 1 Center for Biomedical Informatics, Wake Forest School of Medicine, Winston-Salem, USA; 2 Division of Biostatistics, College of Public Health, The Ohio State University, Columbus, USA; 3 Department of Biomedical Informatics, The Ohio State University, Columbus, USA; 4 Department of Pathology, The Ohio State University, Columbus, USA; 5 Winston-Salem, USA

**Keywords:** Ki67 index, Segmentation, Nuclei detection, Prognosis, Computational efficiency

## Abstract

**Background:**

The Ki67 Index has been extensively studied as a prognostic biomarker in breast cancer. However, its clinical adoption is largely hampered by the lack of a standardized method to assess Ki67 that limits inter-laboratory reproducibility. It is important to standardize the computation of the Ki67 Index before it can be effectively used in clincial practice.

**Method:**

In this study, we develop a systematic approach towards standardization of the Ki67 Index. We first create the ground truth consisting of tumor positive and tumor negative nuclei by registering adjacent breast tissue sections stained with Ki67 and H&E. The registration is followed by segmentation of positive and negative nuclei within tumor regions from Ki67 images. The true Ki67 Index is then approximated with a linear model of the area of positive to the total area of tumor nuclei.

**Results:**

When tested on 75 images of Ki67 stained breast cancer biopsies, the proposed method resulted in an average root mean square error of 3.34. In comparison, an expert pathologist resulted in an average root mean square error of 9.98 and an existing automated approach produced an average root mean square error of 5.64.

**Conclusions:**

We show that it is possible to approximate the true Ki67 Index accurately without detecting individual nuclei and also statically demonstrate the weaknesses of commonly adopted approaches that use both tumor and non-tumor regions together while compensating for the latter with higher order approximations.

## Background

Cell proliferation is the increase in the number of tumor cells due to an imbalance between cell division and cell death or cell differentiation. Cell proliferation is often quantified through Ki67; a nuclear protein that is expressed exclusively during the active cell cycle phases, but not in resting cells in G_0_ [1–3]. Ki67 is widely used in pathology to assess cell proliferation within multiple different neoplasms [1, 4–7]. In breast cancer, Ki67 has shown promise as an independent prognostic marker and as a predictive marker of responsiveness or resistance to chemotherapy or endocrine therapy [8]. The prognostic utility has been also explored in numerous tumor types, most notably in the brain, neuroendocrine, and lymphoid neoplasms, where the Ki-67 proliferation is frequently employed in tumor grading [3].

Controversies exist regarding the prognostic and predictive role of Ki67 mainly due to lack of standardized methods to quantify Ki67 expression [9] and preanalytical methods used during the tissues fixation and slide preparation period. According to the Breast Cancer Working Group, cell proliferation needs to be reported as a *Ki67 Index* that is defined as the percentage of positively stained cells within the total number of malignant cells scored [10]. The recommendations include counting at least 500 and preferably 1000 cells in three randomly selected high-power fields (40×). However, some pathologists consider this method impractical, if not impossible, particularly for small specimens [11]. As an alternative, pathologists often rely on estimating (i.e. eyeballing without formally counting) to approximate the Ki67 Index. Although this technique is less burdensome than formal counting, it often results in significant inter- and intra-reader variability [3].

A working group was assembled from European and North American cancer treatment institutions to devise a strategy to increase the Ki67 Index concordance [12]. In this group, a total of eight laboratories independently computed Ki67 Index for 100 breast cancer cases. Each laboratory director had a track record of publishing one or more peer reviewed articles on the clinical utility of the Ki67 Index. Six out of the eight laboratories used their local protocols to stain one section from a 50 case tumor microarray block using their own standard Ki67 indexing method. The arithmetic average of the Ki67 Index ranged from 15.6% to 31.1, which indicated substantial differences in quantifying this Index across laboratories. Therefore, Ki67 Index calculation achieved only moderate reproducibility across the laboratories among the world’s leading experts. In a follow-up study, 16 laboratories from eight countries calibrated to a particular Ki67 Indexing method and then scored 50 centrally MIB-1 stained tissue microarray cases [13]. The laboratories scored 18 ‘training’ and ‘test’ MIB-1 stained images through a web-based interface for calibration purposes. The laboratory performance showed non-significant but promising trends of improvement through the calibration exercise, underlying the need to standardize the Ki67 Index before its widespread clinical utilization.

In the past 10 years, several automated image analysis techniques have emerged for quantification of the Ki67 Index. In [14, 15], ImmunoRatio, a free cross-platform application for computing Ki67 Index was introduced. ImmunoRatio uses a series of image analysis operations (background correction, color deconvolution, thresholding, segmentation and identification of individual nuclei, and computation of Ki67 positive and negative areas) to approximate Ki67 Index estimation. This estimate is refined by applying a third degree polynomial to map it to the Ki67 Index. However, our analysis shows that fitting a third-degree polynomial does not compensate for the inclusion of non-tumor nuclei in calculations.

Other commercial solutions exist. For example, in [16], Ki67 Index was obtained by counting positive and negative tumor nuclei using a stereology grid. Nuclei detection was accomplished through Aperio Genie/Nuclear algorithms (Leica Biosystems, Buffalo Grove, IL). However, sampling of heterogeneous breast tissue samples using a stereological method is highly prone to under- and over-estimation of Ki67 Index. In [17], Ki67 Index was calculated through a commercially available software: Tissuemorph Digital Pathology (Tissuemorph DP: Visiopharm, Hoersholm, Denmark). The authors suggested that a pathologist should verify the results and make the final decision when computing Ki67 Index using Tissuemorph Digital Pathology. Both Genie and TissueMorph solutions rely on individual cell detection, a process that has a high computational cost considering the size of slides. As a result, Ki67 Index computation takes far longer than how long a pathologist would take to estimate the Ki67 Index.

In this study, we corrected and validated a strategy that does not need the detection of individual nuclei to estimate Ki67 Index accurately. We also statistically demonstrate that Breast Cancer Working Group guidelines can be accurately approximated by computing the area of positive tumor nuclei. Unlike ImmunoRatio based approaches [14, 15] of higher-order polynomials, we determine a linear relationship between the original Ki67 ratio (ground truth) and its approximation by our method. We further show that the error between the approximated Ki67 indices and the ground truth remains relatively unchanged with increasing Ki67 ratios when tested over a reasonable size breast cancer dataset. As a result, the accurate Ki67 Index can be calculated without detecting individual nuclei from Ki67 stained breast cancer images, a process that is computationally expensive and often imprecise.

## Methods

**Fig. 1:**
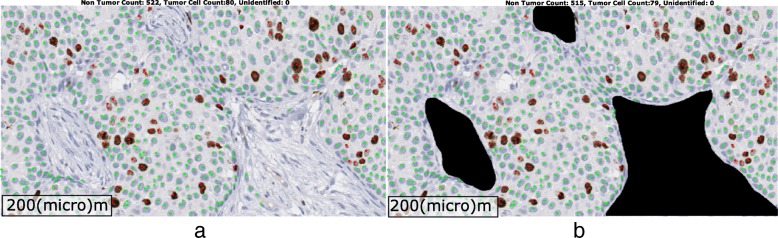
An example image with ground truth overlaid. **a** The tumor positive nuclei are marked with red dots while negatives are marked in green. The non-tumor nuclei were left unmarked. **b** The non-tumor regions are shown in black. These regions were not considered for further analysis. The inclusion of such regions will incorrectly decrease the Ki67 Index because negative nuclei within these regions are abundant

We acquired a dataset of 50 adjacently-cut pairs of Ki67 and H&E whole slide images from 50 different breast cancer patients for this study. Ki67 immunohistochemistry was performed using MIB-1 mouse monoclonal antibody from Dako (Santa Clara, CA) on the Leica Bond III system, 1:400 dilution using high pH retrieval (ER2) for 20 min and the Leica Polymer Refine detection kit. The samples are not publically available and can be made available on request. This study is IRB approved by the Ohio State University, Cancer Institutional Review Board, with Waiver of Consent Process, and Full of Waiver of HIPAA Research Authorization. Furthermore, all samples were fully anonymized by the rules set by the Ohio State University, Cancer Institutional Review Board. All images were acquired at 40× magnification using ScanScopeTM (Aperio, Vista CA) scanner. Following a common practice in pathology, tumor regions were identified on H&E-stained slides and the tumor boundaries were mapped to the corresponding tumor region in the adjacent Ki67-stained slides. First, a board-certified pathologist manually drew tumor boundaries on H&E images which were later transferred over to adjacent Ki67 images. Tumor regions, identified in this manner may still contain some non-tumor regions (stroma and stromal cells, lymphocytes), and there may be non-linear variations due to harsh immunohistochemical staining process. Therefore, a second review was conducted by a pathologist to manually exclude such non-tumor regions from Ki67 images. Figure 1 (b) shows an example image where non-tumor regions were manually removed by an expert pathologist.

The role and the detection of Ki67 could vary according to the breast cancer histology [18]. For this reason, we used three histologic types of breast cancers. We used a total of 50 cases in our experiments. Four of these cases belong to invasive lobular carcinomas, one was invasive tubular carcinoma and 45 were invasive ductal carcinomas. Out of these, 10 were classified as grade I, 22 were identified as grade II, while 18 belonged to grade 3. We selected 75 regions of interest (ROI) images within tumor regions from these 50 Ki67 slides. Each ROI has a size of 1200 × 2300 pixels, approximating a high-power field. The ROIs were selected to represent different concentrations of Ki67 positive nuclei. For the ground truth generation, all nuclei were manually annotated for Ki67 positive and negative. Figure 1 shows an example image where Ki67 positive nuclei are marked with red dots while negative tumor nuclei are annotated in green within tumor regions.

Ki-67 positive nuclei manifest themselves as brown hue in images of breast tissues. The large variations in specimen preparation, staining, imaging as well as true biological heterogeneity of breast tissue often results in variable brown intensities in Ki-67 stained images [3]. These variations affect the accuracy of Ki-67 nuclei segmentation algorithms.

**Fig. 2:**
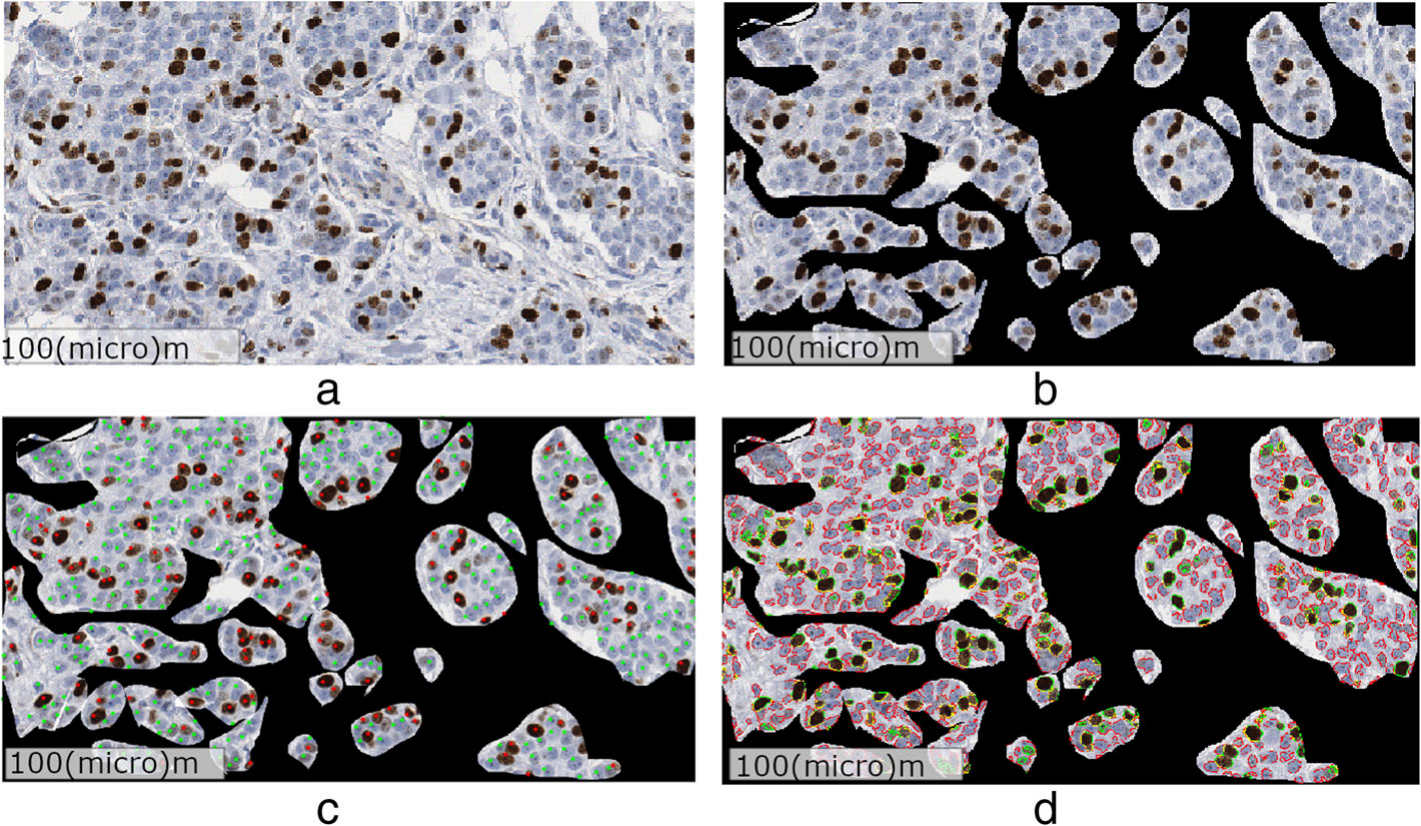
Segmentation results. **a** ROI image containing both tumor and non-tumor nuclei. **b** ROI image after the removal of non-tumor nuclei. **c** Manual annotation of tumor positive and tumor negative nuclei in red and green, respectively. **d** Automatic segmentation of tumor-positive and tumor negative nuclei. The negative tumor nuclei are outlined in red while positive tumor nuclei are outlined in green

We performed nuclei segmentation on Ki-67 stained breast tissue images using an enhanced version of the method we developed in our previous study [3]. Briefly, this method exploits the intrinsic properties of CIE L∗a∗b∗ color space to translate this complex problem into an automatic entropy based thresholding problem. The method in [3] consists of three main components: 1) clustering of RGB color pixels into three clusters based on cluster centroids, 2) color space transformation in the CIE L∗a∗b∗ color space, and 3) entropy thresholding to segment the Ki-67 positive nuclei. The method was designed with an assumption that each image has some Ki-67 positive nuclei. However, there exist situations where Ki-67 positive nuclei are completely absent from an image when the method erroneously starts treating negative nuclei as Ki-67 positive nuclei. To reduce the number of false positives, we modified our previous method to produce correct results for any amount of Ki67 staining. The enhanced version consists of two main steps: 1) an initial segmentation to check if the image contains any Ki67 positive nuclei, and 2) proceed to the methods in [3] if the initial segmentation results in any number of Ki67 pixels. The details of this new method can be found in (M. Khalid Khan Niazi, Y Lin, F. Liu, A. Ashok, M. W. Marcellin, G. Tozbikian, M. N. Gurcan, A. Bilgin: Pathological Image Compression for Big Data Image Analysis: Application to Hotspot Detection in Breast Cancer, submitted). For the sake of completeness, we provide a brief detail about the two main steps in (M. Khalid Khan Niazi, Y Lin, F. Liu, A. Ashok, M. W. Marcellin, G. Tozbikian, M. N. Gurcan, A. Bilgin: Pathological Image Compression for Big Data Image Analysis: Application to Hotspot Detection in Breast Cancer, submitted). During the first step, the method in (M. Khalid Khan Niazi, Y Lin, F. Liu, A. Ashok, M. W. Marcellin, G. Tozbikian, M. N. Gurcan, A. Bilgin: Pathological Image Compression for Big Data Image Analysis: Application to Hotspot Detection in Breast Cancer, submitted) uses two precomputed matrices to assess if an image contains any Ki67 positive nuclei. One of these matrices corresponds to the cluster centroids while the other represents the color transformation matrix. The detail of both these matrices can be found in [3]. The method in [3] is susceptible to false segmentation if an image does not contain any Ki67 positive nuclei. By using precomputed matrices, we are ensuring that we are selecting an image for parameter estimation which contains some Ki67 positive nuclei. These precomputed matrices were computed from an independent dataset of breast cancer consisting of 25 whole slide images. The second step of (M. Khalid Khan Niazi, Y Lin, F. Liu, A. Ashok, M. W. Marcellin, G. Tozbikian, M. N. Gurcan, A. Bilgin: Pathological Image Compression for Big Data Image Analysis: Application to Hotspot Detection in Breast Cancer, submitted) is to take the image which contains at least a few Ki67 positive nuclei and then process it using [3] to compute the actual cluster centroids matrix and color transformation matrix. These new matrices were then used for segmentation of the whole slide images. Figure 2 shows the segmentation results along with the ground truth prepared by an expert pathologist.

Agreement between the proposed area based approximation method and the ground truth was measured using Lin’s concordance correlation coeffficient (CCC) [19] and visualized using Bland-Altman plots [20]. Linear regression was used to estimate the relationship between the proposed method and the ground truth. Statistical analyses were performed using STATA IC 14.2 (StataCorp LLC, College Station, TX).

## Results

### True Ki67 vs. the proposed method

**Fig. 3:**
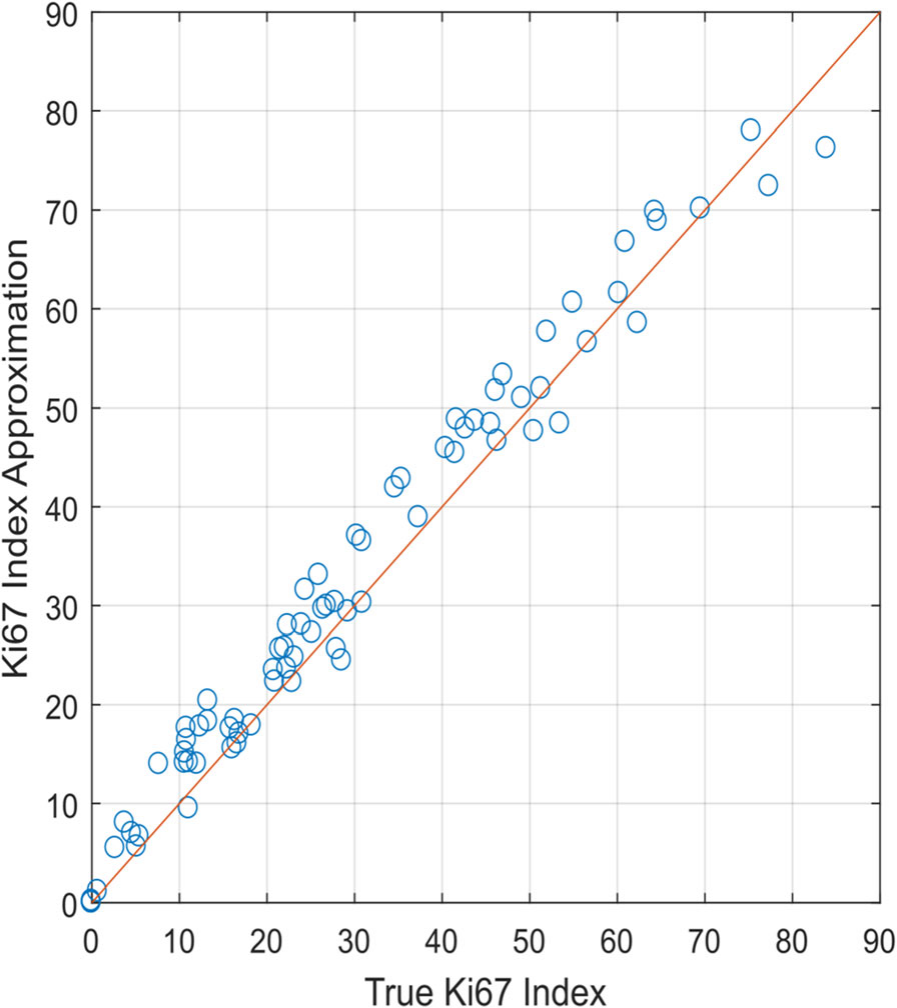
Comparative analysis of True Ki67 Index verses Ki67 Index approximated through area of positive and negative nuclei

The true Ki67 Index of 75 ROI was computed from the manual annotations of Ki67 positive and negative nuclei. Figure 3 plots the true Ki67 Index versus its approximation through area of positive and negative tumor nuclei. The true Ki67 Index is ordered from the smallest to the highest values, to show the wide range of values between 0 and 80%. Because most of the data is above the 45-degree line, the area based method needs to be adjusted to match the true Ki67 Index.

**Fig. 4:**
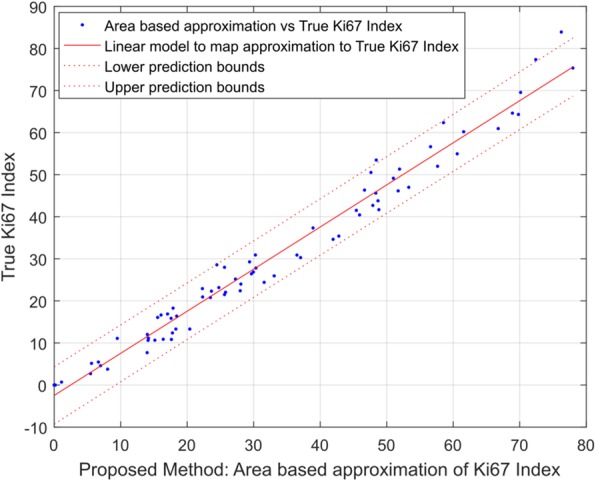
Linear model to map the approximation of Ki67 Index to true Ki67 Index. The model resulted in a root mean square error of 3.339

Figure 4 shows the linear regression model $\check{T},$which maps the Ki67 Index area based approximation (*A*) to true Ki67 Index, T:

1 $$ \left(T\sim \check{T}(A)\right)={c}_1\times A+{c}_2 $$

where the parameter estimates and 95% confidence intervals are as follows: *c*_1 _ = 1.00 (0.96,1.04), and *c*_2_ =  − 2.48 (−3.93, −1.02). The R-square value for the model is 97.46% which shows that the data fits almost perfectly to the model, i.e. to the regression line. The adjusted R-square value for our model is 97.42% with the root mean square error of 3.34.

### True Ki67 index vs. expert pathologist

**Fig. 5:**
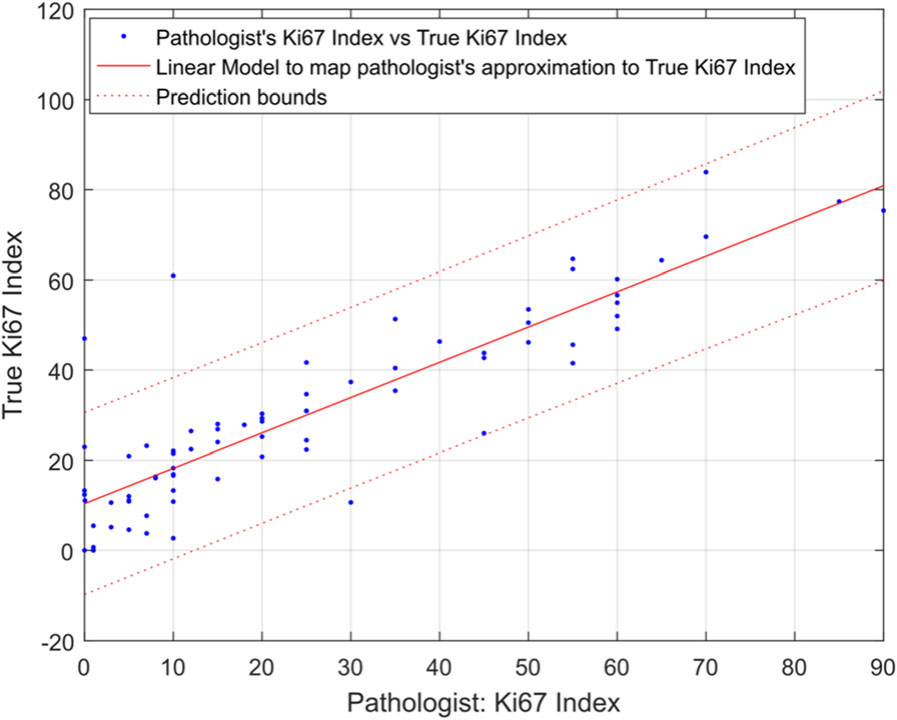
An expert pathologist’s approximation of Ki67 Index vs true Ki67 Index

Figure 5 shows the expert pathologist’s approximation of Ki67 (represented by *P*) Index for our dataset. It also shows a linear model to map *P* to *T*. The R-square value for the model is 77.30% (the adjusted R-square value is 77.00%, root mean square error of 11.21), which is considerably lower than the area based approximation of *T*.

### Ki67 index from the whole slide vs Ki67 index within tumor

**Fig. 6:**
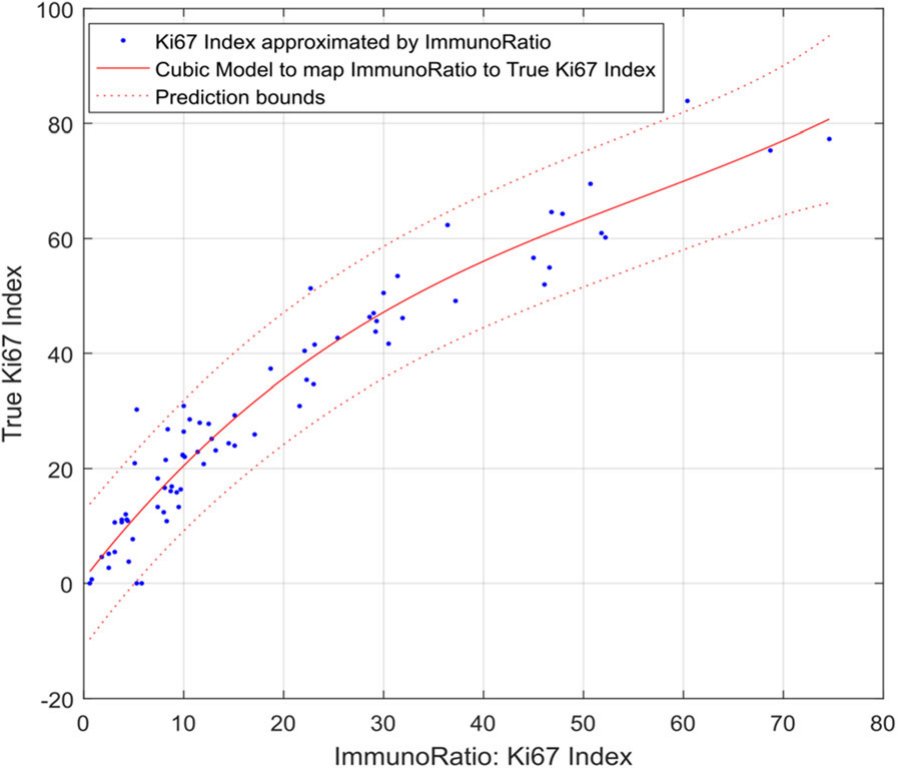
Cubic model’s approximation of Ki67 Index vs true Ki67 Index

We also investigated the effect of carrying out the calculations within tumor regions versus the whole slide. As Table 1 shows a linear regression model only explains 89% of the variability when calculations were performed using the whole slide. According to [14, 15], a third-degree polynomial provides a good approximation to the true Ki67 Index when applied to the whole image (see Fig. 6). Our analysis suggests that a linear approximation of Ki67 Index within tumor region results in a relatively high adjusted R-square value of 97.42%. On the other hand, the cubic model, when applied to the whole image to approximate Ki67 Index, results in a lower adjusted R-square value of 92.65%.

**Table 1:** Statistical summary of different models. Here RMSE stands for root mean square error

Ki67 area based Approximation	R-square	Adjusted R-square	RMSE
Within Tumor (Linear Model)	0.9746	0.9742	3.339
Whole Image (Linear Model) [14, 15]	0.8946	0.8932	6.799
Whole Image (Quadratic Model) [14, 15]	0.9263	0.9243	5.725
Whole Image (Cubic Model) [14, 15]	0.9295	0.9265	5.640

### Statistical analysis

**Fig. 7:**
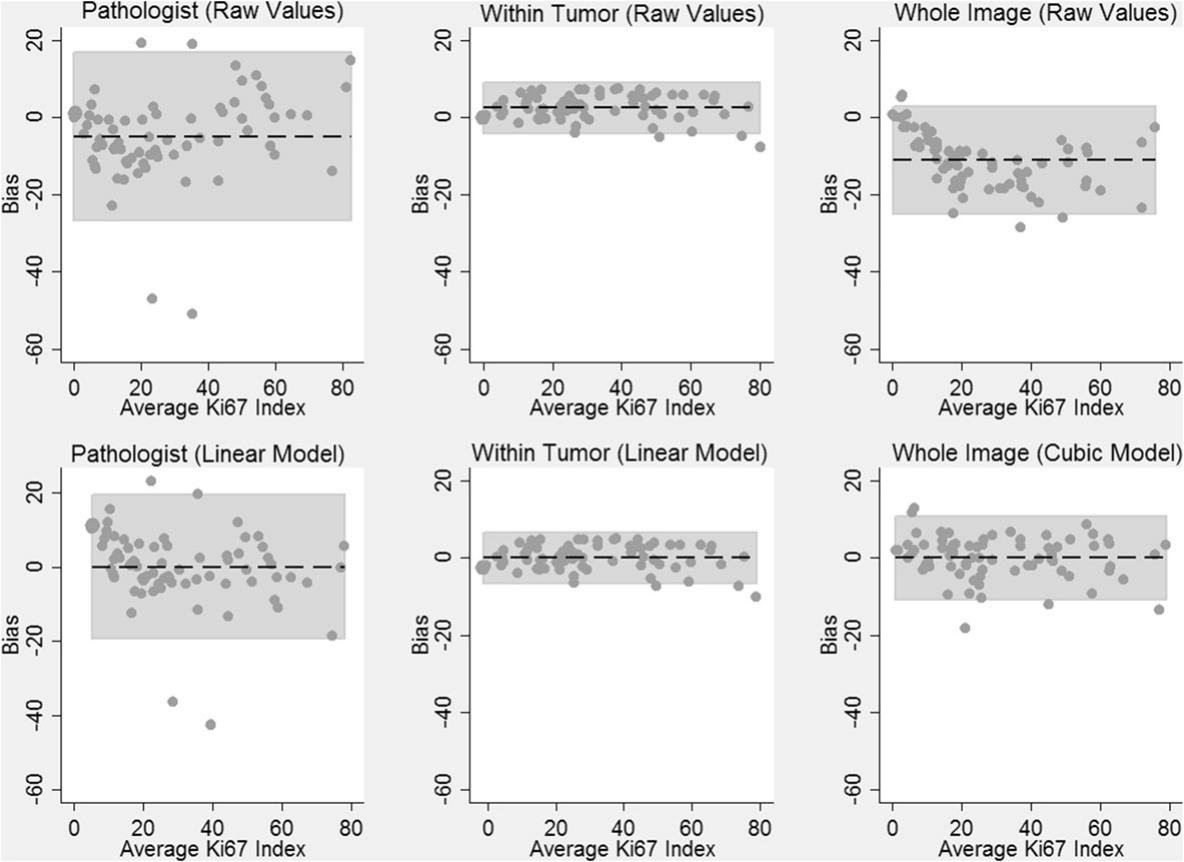
Bland-Altman Analysis Comparing Approximations of the Ki67 Index to Ground Truth. Dashed horizontal lines are the average bias (approximation – ground truth) and the shaded regions are the 95% limits of agreement. Values on the x-axis are the average of the true Ki 67 Index and the approximation

Figure 7 contains the results of Bland-Altman analysis comparing the different approximation methods to the ground truth. Prior to applying the linear model (1), the within tumor approximations exhibited small positive bias (mean = 2.45) and there was no systematic trend in bias with value of the Ki67 index. The limits of agreement of the within tumor approximations were also relatively narrow: (− 4.05, 8.95). After applying model (1), the bias in the within tumor approximations was removed and the limits of agreement remained narrow (− 6.50, 6.50). In contrast, the expert pathologist and whole image approximations were considerably biased (mean = − 4.88 and − 10.83, respectively). Applying a linear and cubic model to these data removed the biases but still resulted in limits of agreement that were much wider than the within tumor approximations: (− 19.44, 19.44) for the pathologist approximations after applying a linear model and (− 10.83, 10.83) for the whole slide approximations after applying a cubic model.

Table 2 contains CCC’s quantifying agreement between each approximation method and the ground truth. The raw Ki67 index values of the area based approximation method exhibited near perfect agreement with the ground truth (CCC = 0.980) and agreement improved slightly after applying Model (1) to account for the small positive bias in the estimates (CCC = 0.987). The approximations made by the expert pathologist exhibited worse agreement with the ground truth (CCC = 0.852) even after correcting for bias using linear regression (CCC = 0.872). The agreement between the whole image approximation method and the ground truth (CCC = 0.798) improved substantially after applying the cubic model (CCC = 0.963), though the level of agreement was slightly worse than what we observed for the within tumor approximations after applying the linear model.

**Table 2:** Concordance Correlation Coefficients (CCC) measuring agreement with ground truth

Ki67 area based Approximation	CCC	95% Confidence Interval
Expert Pathologist (Raw Values)	0.852	(0.780, 0.902)
Expert Pathologist (Linear Model)	0.872	(0.807, 0.916)
Within Tumor (Raw Values)	0.980	(0.969, 0.987)
Within Tumor (Linear Model)	0.987	(0.980, 0.992)
Whole Image (Raw Values) [14, 15]	0.798	(0.726, 0.853)
Whole Image (Cubic Model) [14, 15]	0.963	(0.943, 0.977)

## Discussion

The Ki-67 Index has strong potential to be a significant factor for treatment decision making in breast cancer patients, but it is also one of the hardest to compute [21]. A literature review reveals that many cancer treatment centers across the United States compute Ki-67 Index in a large proportion of tumors from patients with primary breast cancer [22]. This suggests that Ki67 Index is widely used in routine clinical practice although not recommended in national guidelines. Our long-term objective is to standardize the computation of Ki67 Index and systematically review its clinical utility to bring standardization of results among laboratories. The focus of our study was to standardize the computation of Ki67 Index.

In the past few decades, many efforts have been devoted to automating the nuclei detection algorithms in digital pathology [23–31]. However, ever increasing interest in the development of nuclei detection algorithms indicate 1) the complexity of the problem and 2) the inability of current nuclei detection algorithms to provide fast and reproducible results [32]. Moreover, the computational complexity associated with nuclei detection algorithms in histopathology often requires grid computing [33–35] and computationally scalable algorithms [36, 37] to achieve high-throughput image analysis on large size pathology images. Even with these advanced computational methods, the nuclei detection algorithms take far longer than a pathologist’s time to estimate the Ki67 Index. On the other hand, the current method, which only detects positively stained areas without trying to identify individual nuclei, can be combined with grid computing and computationally scalable algorithms, resulting in real-time implementations.

Unlike former studies [14, 15], which established cubic relationships between positively stained areas and the true Ki67 index, our results suggest that there is a linear relationship between the true Ki67 Index and the area ratio of positive nuclei to total nuclei as long as the computation is limited to tumor regions. The value of coefficient *c*_1 _(i.e. *c*_1_ = 1) in Eq. 1 indicates that the area based approximation and the true Ki67 Index only differ by a constant *c*_2_ (*c*_2_ =  − 2.48). However, when non-tumor nuclei are included in computing the Ki67 Index, the true index is harder to predict with simple polynomial models and the estimation error increases. A breast cancer image usually contains subsets of non-tumor nuclei. Although the nuclei sizes might be similar within a subset, they might be completely different across subsets. Apart from the size, these non-tumor nuclei might appear as positive or negative. The amount of non-tumor nuclei may result in an over (or under) estimation of Ki67 Index when the number of positive non-tumor nuclei is higher (or lower) than the negative non-tumor nuclei. Therefore, non-tumor regions need to be excluded from an image before computing the Ki67 Index.

Instead of excluding non-tumor nuclei, the authors in studies [14, 15] suggested using a third degree polynomial to compensate for over- and under-estimation of Ki67 Index. Our study demonstrates (e.g. Table 1) that the amount of non-tumor nuclei, either positive or negative and their variation in sizes are not necessarily governed by a third-degree polynomial. Instead, there is a linear relationship (Eq. 1) to estimate Ki67 Index, with a constant offset of *c*_2_. While we can assume that the size distribution of the positive (and negative) tumor nuclei across different patients is nearly identical to each other, the average sizes of positive nuclei seem to be slightly larger (hence a small *c*_*2*_ value), than those of negative tumor nuclei. Because there is no biological reason for these two different cell groups to have differing average sizes, this small difference can also be explained by the segmentation algorithm. Although it is possible to reduce this difference, hence make *c*_*2*_ close to zero by adjusting the segmentation algorithm, for practical purposes, it will not result in any changes to the Ki67 Index calculation.

The authors of the studies in [14, 15] reported that a third degree polynomial is necessary to map their Ki67 Index with that of the ground truth. Unfortunately, their results were not subjected to a comprehensive statistical evaluation. Moreover, the estimation error after fitting the third-degree polynomial is considerably larger than the estimation error associated with the linear model applied to the area based approximations. While their method is designed to identify individual tumor nuclei, it approximates the pixel area instead of the number of tumor nuclei to estimate Ki67 Index. Our results suggest that, if the tumor nuclei were correctly identified in [14, 15], it should result in a linear relationship between approximated Ki67 Index and the true Ki67 Index.

The comparison of the proposed method with an expert pathologist shows the importance of using image analysis over visual estimation when computing Ki67 Index. Pathologists exhibit considerable differences between visual estimation and true Ki67 Index, suggesting the limitations of the human visual system and resulting perceptual and cognitive challenges they face. Because computers are not affected by these challenges, Ki67 Index computation could be an area where computers can assist pathologists in making accurate decisions.

Throughout the analysis, we relied on an expert pathologist’s annotations for identification of tumor regions. However, a pathologist’s assessment is clearly not without certain limitations. The lack of an automatic method for tumor identification might be a limiting factor in our study.

## Conclusions

This study statistically demonstrates that the Ki67 Index can be approximated reliably by the area ratio of positive tumor nuclei to total tumor nuclei. The linear relationship between the true Ki67 Index and its area based approximation, make it possible to estimate the Ki-67 Index accurately only by calculating the area of stain-positive and negative nuclei within tumor regions. This finding is significant with practical implications because it eliminates the need to detect or count nuclei before computing the Ki67 Index. Our study also demonstrates that the amount of non-tumor nuclei, either positive or negative and their variation in sizes are not necessarily governed by a third-degree polynomial.

In the future, we are planning to systematically review the level of evidence for the Ki-67 Index as a prognostic marker of response to chemo- and hormonotherapy in patients within ER+ tumor to identify patients who are most likely to benefit from chemotherapy. From an image analysis perspective, we are planning to automate the tumor detection process, so that this analysis can be carried out on a whole slide image without any human intervention. ‘In the current study, we have suggested that the Ki67 Index, which is the ratio of positive tumor nuclei to total tumor nuclei, can be approximated through the area ratio of positive to total tumor nuclei. However, in the future we are planning on presenting a method to approximate the number of tumor positive and tumor negative nuclei from the area based Ki67 Index. It is well-known that different institutions produce different staining characteristics, which is one of the reasons, Ki67 calculations cannot be reliably applied across different institutions. As part of our future studies, we will validate our findings on a larger dataset collected from different institutions (to account for slide preparation differences) and validate its generalizability.

## Abbreviations

CCC: Lin’s concordance correlation coeffficient
ROI: Regions of interest
